# Iron in the Symbiosis of Plants and Microorganisms

**DOI:** 10.3390/plants12101958

**Published:** 2023-05-11

**Authors:** Yi Liu, Zimo Xiong, Weifeng Wu, Hong-Qing Ling, Danyu Kong

**Affiliations:** 1 Lushan Botanical Garden, Chinese Academy of Sciences, Jiujiang 332900, China; liuy@lsbg.cn (Y.L.);; 2 Hainan Yazhou Bay Seed Laboratory, Sanya 572024, China; hqling@genetics.ac.cn; 3 State Key Laboratory of Plant Cell and Chromosome Engineering, Institute of Genetics and Developmental Biology, Chinese Academy of Sciences, Beijing 100101, China

**Keywords:** iron, plant symbiosis, iron uptake, iron homeostasis, rhizobium, mycorrhiza

## Abstract

Iron is an essential element for most organisms. Both plants and microorganisms have developed different mechanisms for iron uptake, transport and storage. In the symbiosis systems, such as rhizobia–legume symbiosis and arbuscular mycorrhizal (AM) symbiosis, maintaining iron homeostasis to meet the requirements for the interaction between the host plants and the symbiotic microbes is a new challenge. This intriguing topic has drawn the attention of many botanists and microbiologists, and many discoveries have been achieved so far. In this review, we discuss the current progress on iron uptake and transport in the nodules and iron homeostasis in rhizobia–legume symbiosis. The discoveries with regard to iron uptake in AM fungi, iron uptake regulation in AM plants and interactions between iron and other nutrient elements during AM symbiosis are also summarized. At the end of this review, we propose prospects for future studies in this fascinating research area.

## 1. Introduction

Iron, as one of the most abundant elements on earth, is an essential element for most organisms since it functions as an indispensable co-factor of many enzymes in various crucial metabolic processes [1]. In plants, iron deficiency results in reduced chlorophyll synthesis and photosynthesis, and causes chlorosis and dramatic growth defects. To obtain sufficient iron from soil, plants evolved different strategies for effective iron uptake and homeostasis. The first one is the reduction strategy (Strategy I), which is widely used in all non-graminaceous plants. The second one is the chelation strategy (Strategy II), which is applied by graminaceous plants [2]. Compared with plants, microorganisms engage high-affinity and low-affinity uptake systems for iron uptake [3,4]. The high-affinity uptake pathways include the siderophore-mediated iron uptake pathway and the reductive iron assimilation (RIA) pathway [4]. The low-affinity uptake pathways include the iron-containing protein (e.g., heme, ferredoxin) uptake pathway and the ferrous iron uptake pathway. In general, the high-affinity uptake pathways are adopted by microorganisms when limited iron is available. In contrast, when iron is sufficient, the low-affinity pathways are applied by microorganisms.

Plants and microorganisms in the rhizosphere recruit their own ways to acquire iron from soil until an infection or a symbiosis event takes place between them. In the competition of host plants with pathogens, iron also plays an important role in restricting pathogen growth either by the overaccumulation of iron at the pathogen attack site to induce ROS burst, which leads to the infected cell’s death [5,6], or by withholding iron out of the vicinity of the infection site [2,7].

Symbiosis between a plant and microorganism is a very common phenomenon in nature. In the symbiont, both the plant and microorganism can obtain nutrients from each other, supporting better growth and development. For example, leguminous plants obtain ammonia from symbiotic rhizobia and bacteria obtain carbon compounds from host plants. Similarly, AM fungi supply nitrogen and phosphate to host plants and host plants provide lipids to mutualistic AM fungi [8,9]. Therefore, symbiosis is very important for both mutualistic microorganisms and host plants. In the symbiosis, such as legume–rhizobium and plant–arbuscular mycorrhizal (AM) fungi, iron is coordinated as an important micronutrient for both plants and symbiotic microbes. For legume–rhizobium symbiosis, iron is an essential co-factor for the nitrogenase and the enzymes of the bacterial respiration. A large amount of iron has to be transported from the roots to the bacteroids to support symbiotic nitrogen fixation (SNF). In the last thirty years, many studies focused on this process have been conducted. For the plant–AM fungi symbiosis, recent studies revealed that AM symbiosis can influence the iron uptake of plants. However, the iron transport mechanisms between the plants and AM fungi are still unknown. In this review, we introduce the iron transportation mechanism in the symbiosis and interaction between the plants and symbiotic microbes in terms of the iron uptake, transport and homeostasis.

## 2. Iron Transport in Rhizobia–Legume Symbiosis

Legumes play a crucial role in the nitrogen cycle in agricultural and natural environments since they form symbioses with nitrogen-fixing soil bacteria (rhizobia) that enable the plants to utilize atmospheric nitrogen. Starting with the infection of the legume roots by rhizobia, the symbiotic process eventually forms a new organ, root nodule, where the symbiotic nitrogen fixation occurs. The mature nodule possesses a central infection zone, containing infected and uninfected cells, surrounded by layers of cells termed the cortex [10,11]. Metabolites are transported to the nodule through the vasculature, which terminates in the cortex [10,11]. Nodules are classified as two distinct types, determinate and indeterminate. Determinate nodules, such as those in *Glycine max* (soybean) and *Lotus japonicus*, are spherical without a meristem and embed the infected region in the center surrounded by the cortex layer on the outside [10,11]. Indeterminate nodules, such as those of *Medicago truncatula*, *Pisum sativum* (pea) and *Vicia faba* (broad bean), are elongated or branched shape because they have persistent meristems [11,12]. A mature indeterminate nodule can be divided into at least four zones: zone I is the meristematic region that drives nodule growth; zone II is where rhizobia are released from the infection thread and differentiate into bacteroids; zone III is the site of nitrogen fixation; and zone IV is the senescence zone, where bacteroids are degraded and nutrients are recycled [13]. Once the rhizobia attach to the root hair, they are transported to the cortical cell through an infection thread, and then release into the root’s cortical cells surrounded by the symbiosome membrane (SM), which is a plant-derived membrane [14].

Iron is a crucial micronutrient for nodule development and symbiotic nitrogen fixation (SNF). Symbiotically grown legumes have a particularly high requirement for iron, and iron deficiency severely interferes with their growth and the ability to fix atmospheric nitrogen [15,16]. In soybean, nitrogen-fixing nodules contain approximately 44% of the total iron of the plant, compared to 39% in the leaves, 7% in the seeds and 5% in the roots [17]. To achieve enough iron for nodulation and SNF, the plants and rhizobia mutually stimulate their iron uptake mechanisms. For example, *Sinorhizobium meliloti*, a typical rhizobium, secretes some volatile organic compounds (VOCs), which are able to significantly induce the ferric reductase activity and enhance the rhizosphere acidification [18]. On the other hand, a plant peptide NCR247 secreted by *Medicago truncatula* can be transported into the rhizobium cytoplasm and binds to the haem [19], resulting in a decrease in free haem and the release of the activity of haem-inactivated transcription factor Irr in the rhizobium. Active Irr represses the expression of the *rirA* gene, which encodes a transcriptional repressor of iron-uptake genes [19]. Thus, the secreted NCR247 peptide from host plants will induce the expression of rhizobium iron-uptake genes by binding the haem in the rhizobium cytoplasm [19].

### 2.1. Iron Transport from Root to Nodule

**Figure 1:**
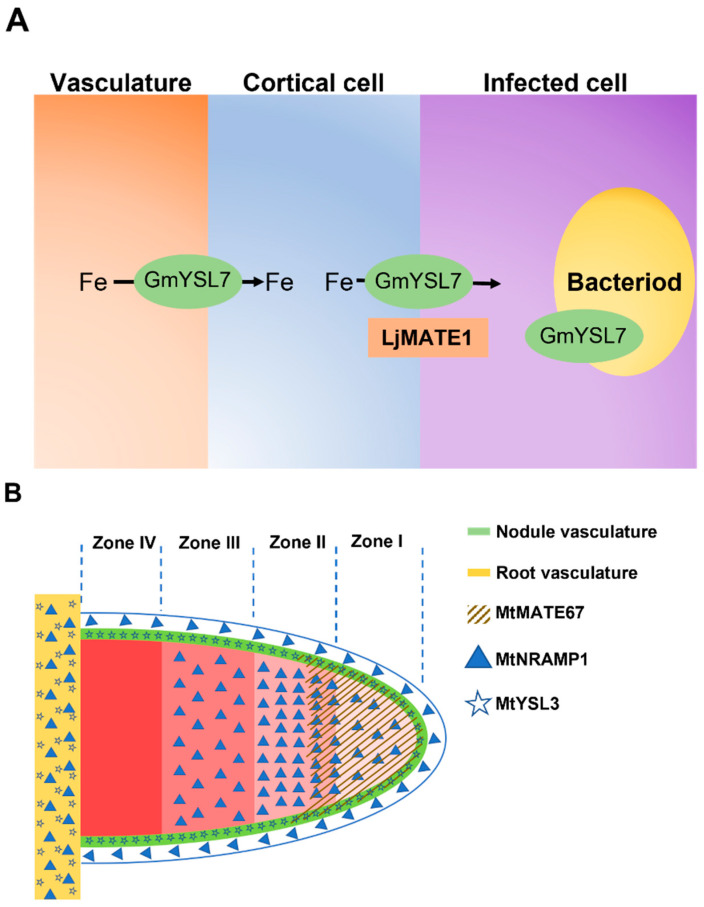
Iron transport from root to nodule. (**A**) Iron transporters in determinate nodule. (**B**) Iron transporters in indeterminate nodule. MtMATE67 is located in zone I and partially in zone II, which next to zone I; MtNRAMP1 is located in root vasculature, cortex, and zone I, II, and III; MtYSL3 is located in both root vasculature and nodule vasculature.

Iron is transported as a form of ferric citrate complex into the xylem of plants [20]. Therefore, iron is also most likely to be imported into the nodule as a ferric citrate complex from the vasculature via the crossing of a number of cell layers to the infected cells [21,22]. Several transporters responsible for iron uptake and transport to nodules have been identified. The citrate transporters, MtMATE67 in *Medicago truncatula* and LjMATE1 in *Lotus japonicus*, which are located in nodules (Figure 1), assist the translocation of iron from the roots to nodules via the transportation of citrate to improve the solubility and availability of iron [23,24]. An iron and manganese transporter MtNRAMP1, a member of the Natural Resistance-Associated Macrophage (NRAMP) protein and located on the plasma membrane of nodules, is mainly expressed in zone II (Figure 1B) and has lower expression level in zone I, zone III, the nodule cortex and root vasculature (Figure 1B). Nitrogenase activity is reduced in MtNRAMP1 knockout mutant *nramp1*-*1*. Exogenous iron supply can rescue the nitrogen fixation to normal levels in *nramp1*-*1* mutant nodules. This indicates that MtNRAMP1 is involved in the iron uptake of the infected cells in nodules [25]. Recently, Yellow Stripe 1-like (YSL) transporters MtYSL3 in *Medicago truncatula* and GmYSL7 in soybean have been demonstrated as being important for iron transport from roots to nodules [26,27]. It was confirmed that GmYSL7 is located in both cortical cells and infected cells, whereas MtYSL3 is only localized in the vascular tissue (Figure 1) [26].

### 2.2. Iron Transport through the Symbiosome Membrane

**Figure 2:**
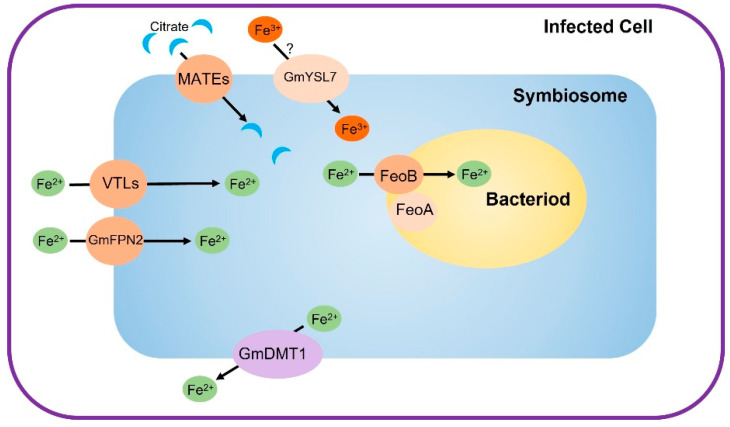
Iron uptake for symbiosome and bacteroid. VTLs and GmFPN2 transport ferrous iron from infected cell cytoplasm into symbiosome, MATEs transport citrate from infected cell into symbiosome, YSLs contribute the transportation of ferric iron from infected cell into symbiosome, FeoAB system transports ferrous iron from symbiosome into bacteroid, GmDMT1 transports ferrous iron from symbiosome to infected cell.

It was proved that the symbiosome enables the uptake of both ferric and ferrous iron by studying isolated symbiosome with a given amount of radiolabeled ferric and ferrous iron [28,29,30]. The uptake of ferrous iron by isolated symbiosome is faster than that of ferric iron [28], indicating that ferrous iron may be the major form transported from the infected cells into the symbiosome. Meanwhile, the transporter for ferrous iron is probably not specific for the acquisition of iron because the transporting efficiency of ferrous iron can be inhibited by copper [28]. The NRAMP family metal transporter GmDMT1 (*Glycine max* Divalent Metal Transporter 1), which is localized on the symbiosome membrane (SM), is able to transport ferrous iron [31], but its transporting direction is still unclear. In the complementary experiment of yeast iron-transport-deficient mutant *fet3fet4*, GmDMT1 was located on the plasm membrane and restored the iron uptake of *fet3fet4* [31]. This indicates that GmDMT1 transports ferrous iron from the extracellular space into the cytoplasm. However, the symbiosome is a vacuole-like structure [32] and the iron uptake into the symbiosome means iron transported out of the cytoplasm. Considering GmDMT1 transports ferrous iron into the cytoplasm in *fet3fet4*, GmDMT1 most likely transports ferrous iron from the symbiosome into the cytoplasm in the infected cells as well (Figure 2) [22,31].

If the symbiosome is considered as an organelle of the infected cell, the transporters that export iron out of the cytoplasm may be involved in iron uptake of the symbiosome. The Vacuolar Iron Transport (VIT) and VIT-like (VLT) proteins are known to transport iron from the cytoplasm into the vacuole [33,34,35]. Two members of the VIT gene family, *GmVTLa* (known as Nodulin-21) and *GmVTLb*, which are highly expressed in nodules [36,37], have been identified by transcriptome data analysis of soybean [38,39,40]. Both GmVTL1a and GmVTL1b are located on the SM of the infected cells (Figure 2), and are able to complement the yeast ferrous iron transport mutant *Δccc1* [37]. Knockdown of *GmVLT1a* and *1b* causes defected nodule development and reduced iron content in nodules and bacteroids [36]. The GmVTL1a homologues in *Medicago truncatula*, MtVTL4 and MtVTL8, are also expressed in nodules and MtVTL8 is localized on the SM [41]. Both MtVTL4 and MtVTL8 can rescue the yeast *Δccc1* mutant, indicating that they are ferrous iron transporters. Expression of *MtVTL8* alone reverted the defect phenotype of nodule development in the 13U mutant, which has a 30 kb deletion spanning *MtVTL4* and *MtVTL8* in the genome, while expression of *MtVTL4* did not [41]. Further analysis of the iron contents in the 13U and *vlt4* mutants revealed that the iron content of the rhizobium in the mutant roots was significantly lower than that of the wild type, while the iron content in the cytoplasm of the infected cells remained similar to that of the wild type. These results indicate that MtVTL4 and MtVTL8 are specifically required for iron delivery into the bacteroids [41]. Another VIT protein of *Lotus japonicus*, LjSEN1, an important regulator of rhizobia differentiation in the nodule [42], was suggested to be an iron transporter in the infected cells as well [22,37]. However, there is still no direct evidence to show whether LjSEN1 is located on the SM.

Another type of ferrous iron transporter mediating iron out of the cytoplasm into the organelles is ferroportins [43]. *Medicago truncatula* nodule-specific gene *Ferroportin 2* (*MtFPN2*) encodes an iron efflux protein located on the SM of the infected cells (Figure 2) and the vascular intracellular compartments [44]. Mutation of *MtFPN2* resulted in a severe reduction in nitrogenase activity, and the mislocalization of iron in the nodules [44]. This suggests that MtFPN2 is indispensable for iron transport into the symbiosome.

As well as ferrous iron, a symbiosome is able to uptake ferric iron [29,30]. It was found that the iron-activated citrate transporter MtMATE67 is located not only on the plasma membrane of nodule cells but also on the symbiosome membrane in *Medicago truncatula* [23] (Figure 1B). The loss function of *MtMATE67* resulted in the accumulation of iron in the apoplasm of nodule cells. MtMATE67 is responsible for citrate efflux from nodule cells into the symbiosome to ensure the solubility and mobility of ferric iron in the apoplasm and further uptake into nodule cells [23]. Based on the fact that MtMATE67 itself does not transport the iron–citrate complex, there must be another system for iron translocation. MtFPN2 or MtVTL8 may be good candidates since these two ferrous transporters are located on the SM [22,44]. Considering that MtFPN2 or MtVTL8 only transport ferrous iron, a ferric reductase is needed to reduce ferric iron to ferrous iron before the iron can be transported by MtFPN2 or MtVTL8. Another possibility is that there is an unknown ferric iron transporter working with MtMATE67 on the SM.

Yellow Stripe 1 (YS1) is induced by iron deficiency and transports the Fe^3+^-DMA (deoxymugineic acid) complex in maize (*Zea mays*) [45]. Yellow Stripe 1-like (YSL) transporters also play a significant role in plant iron homeostasis. In soybean, GmYSL7 is localized on the plasma membrane of cortical cells in nodules and the SM of the infected cells (Figure 1A and Figure 2) [26,46]. Its iron uptake activity (for both ferric and ferrous) was proved by complementary tests of yeast mutants *fet3fet4* and *Δccc1*. Furthermore, it was demonstrated that GmYSL7 as an iron transporter preferentially transports chelated iron (both ferric and ferrous iron) [26].

### 2.3. Transporting of Iron into the Bacteroids

Free rhizobia in soil need to manage iron uptake from an oxidizing environment. Some transporters in free-living rhizobia have been well studied, such as the TonB-ExbBD complex and ABC (ATP-binding cassette-type) transporters, which transfer ferric siderophore and the heme into rhizobia. However, the expression of TonB-dependent receptors, TonB and ABC transporters, are down-regulated in the bacteroid, and neither the rhizobia mutants of heme transporters or the mutants of TonB/ExbB/ExbD have a defect in symbiosis or nitrogen fixation [47,48,49,50]. These results suggest that the major way that the bacteroid obtains iron is different from free rhizobia [11]. It is worth noting that an isolated bacteroid exhibited ferrous iron uptake activity [28,30]. A FeoAB system, which transports ferrous iron, has been reported in pathogenic facultative anaerobic bacteria [12,51]. FeoB is a ferrous iron transporter widely distributed among bacteria and archaea, while *feoA* encoding an auxiliary protein which is necessary for ferrous iron uptake [52]. *FeoA* and *FeoB* are located in the same expression operon [52]. The incubation of soybean roots with the *feoA* or *feoB* deletion strains led to small and ineffective nodules with few bacteria and compromised nitrogen fixation activity [53]. A mutant strain *E40K*, which carried a missense mutation within the *feoA* gene, exhibited a diminished iron uptake activity, although it is able to develop nodules with nitrogen fixation activity in soybean [53]. This character of *E40K* makes it possible to evaluate the function of the FeoAB system under the state of symbiosis. The measurement of ^55^Fe^2+^ uptake by isolated bacteroids illustrated that the *E40K* strain had lower uptake activity than the wild type [53]. These studies indicate that the FeoAB transport system is responsible for ferrous iron uptake into bacteroids.

### 2.4. Iron Homeostasis in Rhizobia–Legume Symbiosis

As the most important function of nodules, symbiotic nitrogen fixation (SNF) requires a large amount of iron. It was shown that iron deficiency significantly inhibits nodule development and nitrogen fixation [54]. To meet the increased requirement for iron, legumes stimulate the iron-deficiency-induced responses in roots to obtain more iron from soil [55], such as secreting more protons and reductants [56,57], up-regulating the activity of the ferric chelate reductase [58,59], and the accumulation of H-ATPase and IRT1 proteins around the cortex cells of nodules [60]. The bHLH (basic helix–loop–helix) transcription factors play crucial role in the regulation of the iron deficiency response [61,62,63,64]. GmbHLH300 is remarkably up-regulated in the nodules of the *Gmysl7* mutant and *GmYSL7OE* plants [26]. Meanwhile, the expression of *GmbHLH300* was induced under both low and high iron conditions, suggesting that it plays a crucial role for iron homeostasis in soybean nodules. Wu et al. also found that GmbHLH300 negatively regulates the expression of *GmYSL7* and *ENOD93*, which are two positive nodulation regulator genes [26].

## 3. Iron in Arbuscular Mycorrhizal Symbiosis

Arbuscular mycorrhizal (AM) symbiosis is more popular in nature compared with rhizobia–legume symbiosis since 80% of plants growing under natural conditions are associated with mycorrhizae [55,65,66]. AM fungi provide plants with essential mineral nutrients, such as phosphorus (P) and nitrogen (N), and fungi obtain their carbon from the host plants in the form of plant photosynthates and fatty acids in return [67,68,69,70]. Additionally, AM fungi can improve the biotic and abiotic stress tolerance in plants. It has been reported that AM symbiosis can both increase and decrease the iron uptake of host plants [55,71]. A meta-analysis of 233 studies supports that there is a positive impact of AM fungi on crop plants’ iron nutrition [72]. Studies showed that the impact of AM fungi on iron uptake is dependent on the growth conditions, host plant species and AM fungi species. Using ^59^Fe as a tracer, Caris et al. found that the iron uptake of *Glomus mosseae* (an AM fungus)-cultured sorghums (Strategy II plant) increased in calcareous iron-deficient soil, but the iron content of peanut (Strategy I plant) had no change [73]. This result suggests that AM fungi increase the iron uptake in Strategy II plant but not Strategy I plant. However, Kabir et al. discovered that AM fungi increase the iron uptake in sunflower (Strategy I plant) when the plants are inoculated with a mixed endomycorrhizal spore including *Glomus intraradices*, *Glomus mosseae*, *Glomus aggregatum* and *Glomus etunicatum* [74]. On the other hand, the AM plants grown at a low pH showed higher iron uptake than those grown at a high pH [75,76]. As well as pH, the temperature influences the iron uptake of the host plant in AM symbiosis. The sorghum inoculated with *Glomus macrocarpum* (a vesicular-arbuscular mycorrhizal fungus) had more than 10-fold iron grown at 25 °C or 30 °C than that grown at 20 °C [77]. Al-Karaki et al. found that water stress promoted the iron uptake in AM-fungi-cultured wheat [78,79]. All of the above studies indicate that AM symbiosis increases the iron uptake of host plants in some certain conditions. Nevertheless, further understanding on how the growth conditions, host plants and AM fungi species affect the iron uptake of the AM plant is still lacking.

### 3.1. Iron Uptake in AM Fungi

In AM roots, AM fungi develop arbuscules to facilitate nutrient exchange with the host plants. In the soil, AM fungi develop extensive and highly branched external mycelium to absorb nutrients beyond the depletion zone that develops around the roots [80]. In this way, the AM plants can absorb nutrients through both the plant roots and mycorrhiza [80]. Caris et al. found that providing ^59^Fe to the hyphae resulted in a radioactive signal appearing in the shoots of host plant in both peanut and sorghum [73]. This result indicated that iron was delivered from the AM to the host plant. Kobae et al. performed similar isotopic tracing in maize and observed the same result [81].

Only a few iron transporters in AM fungi have been identified so far. Genome sequencing and transcriptomic analyses of *Rhizophagus irregularis*, an arbuscular mycorrhizal fungus, revealed two potential iron permeases, RiFTR1 and RiFTR2, which are expressed in germinated spores and mycorrhizal roots [82]. RiFTR1 is up-regulated (10-fold) during the symbiotic phase of the fungus, which indicates that RiFTR1 plays an important role in the biotrophic phase of AM fungus [82]. The genes encoding NRAMP family members *RiSMF1*, *RiSMF2*, *RiSMF3.1* and *RiSMF3.2* were characterized in *Rhizophagus irregularis*. In the complementary experiment of yeast *fet3fet4* mutant, only *RiSMF3.2* was proved to be an iron transporter [83].

### 3.2. Iron Uptake Regulation in AM Plants

The research on the iron uptake regulation in AM symbiosis is relatively lacking compared with that of rhizobia–legume symbiosis. Recently, transcriptome analysis in the wheat roots colonized by AM fungus revealed that two iron–phytosiderophore transporters were up-regulated during AM symbiosis [84], indicating that wheat may secrete more siderophores in the symbiosis with AM fungus. On the other hand, in a genome-wide analysis of the nodulin-like gene family in bread wheat, six *VIT* subfamily genes were found to be down-regulated in response to AM inoculation, and five of them were significantly down-regulated only at the fully colonized stage [85]. These results suggest that iron might be involved in the AM symbiosis process.

Some evidences have provided clues on the molecular mechanism of AM fungi positively alleviating iron deficiency for host plants. In Strategy I plants, AM fungi resulted in a significant improvement in iron concentrations in the roots and the shoots of sunflower under iron deficiency [74]. In the presence of AM fungi, the expressions of transport genes *HaIRT1* and *HaNramp1* and the ferric reductase gene *HaFRO1* were up-regulated and the ferric reductase activity was increased as well [74]. Similarly, increased siderophore release was observed in AM fungi symbiosis with *Tagetes patula nana* [86]. In *Medicago sativa* L., the expression of ferric iron reductase gene *MsFRO1* is significantly induced in roots by cultivation with AM fungi under iron-deficient conditions [87].

In Strategy II plants, Prity et al. reported that phytosiderophore (PS) release was increased in the sorghum root cultured with AM fungi, and the expressions of iron uptake related genes *SbDMAS2*, *SbNAS2*, and *SbYS1* were elevated under iron deficiency, compared to the root without AM fungi [88]. Sulfur deficiency had a strong, negative impact on the Strategy II iron acquisition, causing a reduction in iron concentration and induced *ZmNAS1* and *ZmYS1* expression in the roots in non-mycorrhizal maize [89]. However, in mycorrhizal plants, the iron content and the expression of *ZmYS1* remained at a normal level during sulfur depletion, and the expression of *ZmNAS1* was down-regulated [89]. These results imply that the maize with AM symbiosis maintained sufficient iron during sulfur depletion. Chorianopoulou et al. suggested that iron is mainly transported directly to the root from the AM fungi via a special symbiotic iron uptake pathway [89]. This result is consistent with the observations of Caris et al. and Kobae et al. using isotopic tracing which were mentioned before [73,81]. It was also found that the transcript levels of oligopeptide transporter genes *ZmOPT8a* and *ZmOPT8b* in mycorrhizal roots were induced 194- and 62-fold, respectively, than non-mycorrhizal roots [81]. However, the expression levels of other iron-uptake-related genes such as *ZmDMASa*, *ZmYS1*, *ZmIRTa*, *ZmIRTb* and *ZmIRO2a* were similar between non-mycorrhizal roots and mycorrhizal roots [81]. Considering the rice OsOPT1, OsOPT3, and OsOPT4 transport ferric–nicotianamine complex [90], ZmOPT8 is probably involved in iron transport in the mycorrhizal root [81].

### 3.3. Interactions of Iron and Other Nutrient Elements during AM Symbiosis

It was reported that iron interacts with other mineral elements during uptake and homeostasis in plants including AM plants [91]. Phosphorus (Pi) was proven to have a negative effect on the iron uptake of AM plants [92]. Iron uptake decreased under a high Pi condition, whereas it increased under a low Pi supply in AM plants [93]. AM fungi release significant amounts of organic acids to mobilize the phosphorus bounded to iron oxides in soil and increase the Pi acquisition of host plants [94]. There is a possibility that iron is also mobilized by the organic acids under a low Pi supply and are easier to absorb by host plants. The effect of iron on Pi acquisition is barely known. Recently, Pang et al. discovered that AM fungi enhanced Pi uptake when FePO_4_ was used as Pi sources but not KH_2_PO_4_ [95]. In addition, co-inoculation of AM fungi and rhizobia enhanced nodulation under the use of FePO_4_, but not KH_2_PO_4_, as a Pi source [96].

Zinc is another nutrient element that has been revealed as interacting with iron in AM plants. In sunflower, the uptakes of iron and zinc were both up-regulated under iron deficiency in AM plants [74]. This is consistent with the expression of *HaZIP1*, responsible for Zn uptake, being significantly up-regulated following AM fungi inoculation under Fe deficiency [75]. Ibiang et al. reported that zinc treatment leads to the iron accumulation in roots in AM soybean [96]. However, excess zinc increases the iron content in fruit and decreases iron in mycorrhizal roots in AM tomato [97].

## 4. Prospects

Although the information with regard to iron in AM symbiosis is limited, it is meaningful to discover that AM symbiosis plays positive roles in the relief of iron deficiency for plants since AM symbiosis is popular in crops suffering from iron deficiency such as sorghum, maize, potato and peanut. This means it is possible to relieve iron deficiency in crops by applying AM fungi in soil. However, the mechanisms involved in this process need to be studied in the future. For instance, AM fungi induce the expression of the iron-uptake-related genes of host plants under iron deficiency. In this process, the signal transport between AM fungi and the host plant needs to be investigated to answer how AM fungi regulate the host’s gene expression. In the meantime, studies have shown that plants can obtain iron from AM fungi directly, but how iron is transported from AM fungi into plants is not clear.

In the past decades, researchers have made remarkable progress in understanding the mechanisms of iron transport and distribution in rhizobia–legume symbiosis. However, some questions still remain to be accomplished to illustrate the overall picture for iron homeostasis in rhizobia–legume symbiosis. Firstly, GmYSL7 is located on the SM in the infected cells. However, neither its function for iron transport through the SM, or the transport direction of iron on the SM have been demonstrated (Figure 2). Secondly, the impact of the interaction of the host plants and the symbiosis bacteria on the regulation of iron homeostasis is still not clear. Thirdly, in the high iron environment such as the symbiosome and the bacteroid, the iron efflux and storage mechanisms are crucial for avoiding iron toxicity. It will be meaningful to figure out the detoxification mechanisms. Actually, there are some clues for iron efflux from the symbiosome and the bacteroid. For instance, Wittenberg et al. showed that the bacteroids released a large amount of siderophores-bound iron into the symbiosome space in isolated soybean nodules [98]. GmDMT1 was suggested to transport iron from the symbiosome to the cytoplasm [31]. These results indicate that the iron level is regulated by an unclear system in the symbiosome. Recently, GmbHLH300 has been shown to be involved in the regulation of iron homeostasis in the infected cells. More investigation of the GmbHLH300 network may reveal unknown details for iron detoxification in the symbiosome.

## Data Availability

Not applicable.
